# The post-pandemic legacy: the breakthrough of digital health and telemedicine

**DOI:** 10.1093/cvr/cvab178

**Published:** 2021-06-18

**Authors:** Nico Bruining

**Affiliations:** Digital Cardiology, Department of Clinical Epidemiology and Innovation, Thoraxcenter, Department of Cardiology, Erasmus MC, Room Na-312, Dr. Molewaterplein 40, 3015 GD Rotterdam, The Netherlands

**Keywords:** Telemedicine, Remote monitoring, Digital Health, COVID-19, mHealth

The ongoing pandemic is taking a huge toll on our healthcare systems, requiring us to adapt promptly. One of the most alarming health-associated complications we experienced was the reduction in mainstream care and that patients evade care. Telemedicine seems to solve problems such as social distancing, contact with between caregivers, distributing patient data, medical teaching, and sharing scientific results (webinars and virtual congresses). Further digital advancements which could improve medicine have been high on the wish list for years, but major breakthroughs were not yet in sight. The pandemic now seems to bring change.

Today, we can keep in touch with our loved ones and friends anytime, anywhere through our smartphones. We order goods and food via apps and you can even travel the world with your smartphone. Booking, saving your tickets, navigation, communication, and payment are all possible with one device. They are also developing towards more health-oriented and in combination with sensor-loaded smartwatches/fit bits you can monitor your fitness level and take measurements such as limited electrocardiogram (ECG) recordings and monitor your oxygen saturation (important with COVID-19). Given such developments, which have a major impact on our daily life, remarkably, we are not yet applying them on a large scale in healthcare!

These technical advancements in digital health and the high demand for telemedicine during the pandemic are a renewed impulse for telemedicine. A brief glimpse into the past learns they made the first remote diagnosis in 1897.^1^ In the middle of the night, a mother suspected her baby might have croup and asked for help by phone. The doctor asked the mother to hold the child up to the microphone so that he could listen to the cough. His diagnosis was that this was not croup and that the family could go to sleep peacefully. The recognized father of telemedicine as we know it today is Willem Einthoven (1806–1927) who sent an ECG by telephone in 1905. Since then, it has taken many decades for further progress to be made. The start of the digitization of healthcare in the early 1970s, together with the arrival of the Internet, has led to new technical possibilities and renewed interest. However, acceptance and implementation have been slow since then as there were quite several challenges to overcome, such as:^2^

- Costs-effectiveness and reimbursement.
- Technical issues and implementation in the existing workflow.
- Acceptance by both patients and caregivers.

More recently, there is growing evidence of the clinical benefits of telemedicine and telecardiology.^3^ Because of the pandemic, this is now moving much more rapidly because it is high on the agenda of many stakeholders, including politicians. However, there is a fear that if the pandemic ends, we will soon return to our old habits and customs and not persist. As a medical community, it is now our job to use the current momentum to continue to develop and implement these coveted digital tools. A few years ago, the European Society of Cardiology (ESC) recognized the growing importance of digital health, took action, and presented a roadmap. Part of this was the installation of a special Digital Health Committee (DHC). Coincidentally, just before the pandemic, DHC chair Prof. Martin Cowie presented a commentary entitled ‘Is the digital revolution the beginning of a golden age or just the next fad?’ with an emphasis on key areas of digital health.^4^ It looks like the pandemic will answer this question.

We are currently seeing a deluge of innovations around the use of digital health and telemedicine related to COVID-19 in the literature and would like to introduce some of them to you. Neubeck *et al.*^5^ present a review of studies looking at the impact of social distancing and quarantine on cardiovascular patients, showing, as expected, a significant negative effect. Further research shows that the use of telemedicine can well compensate for these negative aspects. They conclude that further development of tools and implementation in existing care systems is necessary. We often think that telemedicine is to be in contact with the outside world only, but it also proves to be very useful within the hospital itself, as described by Alkhouli *et al.*^6^ in ‘Will the COVID-19 Epidemic Reshape Cardiology’. They report that in-hospital telemedicine is helpful to protect caregivers from direct contact with infected patients and speeds up peer consultations that do not require direct personal contact.

As described earlier, the field of mobile devices and health (mHealth) is undergoing rapid development. Its applications and use are incredibly useful under the current circumstances. Leite-Moreira *et al.*^7^ describe the potential for patient education, prevention, and patient management. Another excellent mHealth application/program very useful during the current pandemic is Telecheck-AF.^8^ It provides patients suspected of atrial fibrillation the option for a teleconsultation during which they can perform a heart rate and rhythm monitoring online with their cardiologist. This app, developed by Maastricht University, is available free to other institutions and patients during COVID-19. Recently, the results of a large-scale multi-centre study have been published.^8^

An important milestone in the ESC's digital roadmap was the recent launch of *The European Heart Journal—Digital Health* (https://academic.oup.com/ehjdh), dedicated to connecting the clinic with digital health technology advancements. It published recently two remarkable studies related to COVID-19: (i) Maurizi *et al.*^9^ presented ‘Use of smartphone-operated ECG for home ECG surveillance in COVID-19 patients’ and (ii) Shah *et al.*^10^ ‘Antiarrhythmic Drug Loading at Home Using Remote Monitoring: A Virtual Feasibility Study During COVID-19 Social Distancing’. The titles speak for themselves and both articles show not only the current benefits of digital health and telecardiology but also those for the future.

Digital health was the past couple of years already in the spotlight, and we all agree that further digitization is essential for medicine. The pandemic showed that digital tools, such as telemedicine, were essential to continue care. As we now have momentum, it is our duty to move forward with the digital agenda and to prepare ourselves for the future.

**Conflict of interest:** none declared.

## Figures and Tables

**Figure:**
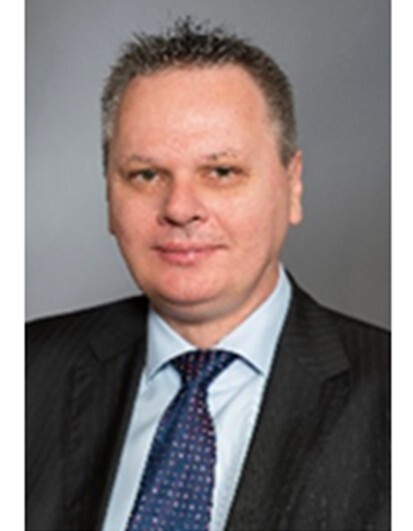


## Author

**Biography:** Nico started his career in 1985 at the Thoraxcenter (Department of Cardiology, Erasmus MC, Rotterdam, The Netherlands) in the catheterization laboratory with some of the pioneers in interventional cardiology, Professor’s Patrick Serruys and Pim de Feyter. In 1993, he joined as a scientist the Department of Clinical and Experimental Information Processing (Now Digital Cardiology) of the Thoraxcenter, of which he is currently the head. His interest lies in the innovative use of computer technology to improve cardiovascular care. His prior research involved the use of cardiovascular imaging and processing to evaluate novel treatment approaches. It involved him in the early developments of three-dimensional (3D) echocardiography, culminating in 1998 with the defense of his thesis—“Quantitative 3D echocardiography of the heart and coronary vessels” under the supervision of Prof. Jos Roelandt. His current field of interest is Digital Health, and he is the inaugural editor-in-chief of the *European Heart Journal—Digital Health*. Nico is a fellow of the European Society of Cardiology (ESC), vice-chair of the ESC's Digital Health Committee, member of the ESC Communication and Congress Program Committee’s and chair of the EAPCI's Innovation and Digital Cardiology Committee.
